# Heavy Metal Contamination Assessment and Partition for Industrial and Mining Gathering Areas

**DOI:** 10.3390/ijerph110707286

**Published:** 2014-07-16

**Authors:** Yang Guan, Chaofeng Shao, Meiting Ju

**Affiliations:** College of Environmental Science and Engineering, Nankai University, Tianjin 300071, China; E-Mails: kiranedved@yeah.net (Y.G.); jumeit@nankai.edu.cn (M.J.)

**Keywords:** environmental assessment, soil heavy metal contamination, geo-accumulation index, Nemerow index, industrial and mining gathering area

## Abstract

Industrial and mining activities have been recognized as the major sources of soil heavy metal contamination. This study introduced an improved Nemerow index method based on the Nemerow and geo-accumulation index. Taking a typical industrial and mining gathering area in Tianjin (China) as example, this study then analyzed the contamination sources as well as the ecological and integrated risks. The spatial distribution of the contamination level and ecological risk were determined using Geographic Information Systems. The results are as follows: (1) Zinc showed the highest contaminant level in the study area; the contamination levels of the other seven heavy metals assessed were relatively lower. (2) The combustion of fossil fuels and emissions from industrial and mining activities were the main sources of contamination in the study area. (3) The overall contamination level of heavy metals in the study area ranged from heavily contaminated to extremely contaminated and showed an uneven distribution. (4) The potential ecological risk showed an uneven distribution, and the overall ecological risk level ranged from low to moderate. This study also emphasized the importance of partition in industrial and mining areas, the extensive application of spatial analysis methods, and the consideration of human health risks in future studies.

## 1. Introduction

The heavy metal contamination of soil has caused widespread concern in the international and domestic communities [1,2,3]. The major sources of soil heavy metal contamination are industrial and mining activities [4,5]. Statistics show that over 10 million hectares of land in China are threatened by heavy metal contamination, with some 2 million hectares being mining areas. The heavy metal contamination of soil poses a serious threat to the quality and safety of the agricultural and ecological environment in China [6,7,8]. According to the Twelfth Five-Year Development Plan for Environmental Protection and the National Environmental Protection Twelfth Five-Year Plan for Technology Development, the prevention of soil heavy metal contamination is a key task for China, and typical industrial and mining areas are key areas of soil contamination studies. The Soil Environmental Protection and Comprehensive Management Arrangements recently issued by China’s State Council point out that partition assessment and the management of contaminated soil are imminent for typical areas, including industrial and mining areas in China. 

In the interest of preventing soil heavy metal contamination caused by industrial and mining activities, local and international scientists and engineers have studied heavy metal contamination in different regions. By determining the total concentrations and chemical speciation of toxic metals, Zhou *et al.* [4] studied soil contamination in the vicinity of the Dabaoshan Mine, Guangdong Province, China, and found that the environmental pollution in this area over the past decades has been caused by the contamination of a combination of Cu, Zn, Cd, and Pb. After further analysis, the authors predicted that the potential environmental risk caused by these metals would increase with time. Li *et al.* [9] conducted a field survey to investigate the metal and As contamination levels in the soil and vegetables in four villages located along Baiyin, China and evaluated the possible health risks posed by such contamination to the local population through the food chain. The results revealed the most significantly contaminated soil in every village, the degree of contamination of vegetables in the four villages, and the environmental health risks assessed through breathing rate and food intake. Wang *et al.* [10] studied the distribution of 10 heavy metals in the agricultural soil surrounding the world’s largest Sb mine, located in China, and explored the degree and spatial distribution of the heavy metal pollution of the Sb mine-affected agricultural soil. The results showed large amounts of 10 heavy metals as well as integrated pollution indexes. Luo *et al.* [11] investigated the metal pollution in the surrounding environment of an inoperative e-waste processing facility and found that the soil at former incineration sites had the highest concentrations of Cd, Cu, Pb, and Zn and that the uncontrolled e-waste processing operations caused serious pollution to the local soil and vegetable products. The cleaning up of former incineration sites should be thus a priority in any future remediation program. Zhao *et al.* [12] investigated heavy metal contamination near the Dabaoshan mine and found that the average concentrations of Cu, Zn, Cd, and Pb in the soil in the study area are all above the natural soil background levels, with Cd being the major pollutant that poses a human health risk. Fu and Wei [13] conducted a survey in an old Sb mine in Xikuangshan, China, and found high concentrations of Hg and Cd, moderate concentrations of As, Pb, and Zn, and Cr concentrations comparable to the background values. 

The concentration of heavy metal contaminants in soil and the spatial variations of heavy metal contaminants have been the focus of previous studies. Comprehensive analysis methods have also been utilized in studies on soil heavy metal contamination. At present, the integrated pollution index is the most widely used method in many studies, such as that by Wang *et al.* [10]. With Pollution-free vegetable production environment requirements (GB/T 18407.1-2001) [14] as basis for evaluation and by using the Nemerow index method, Xu and Ji [15] assessed the mean integrated heavy metal pollution index of the soil in Jiangsu province. Zhao *et al.* [16] applied the geo-accumulation index (I_geo_) and the pollution load index to assess environmentally sensitive elements in Xining, China. Shi *et al.* [17] evaluated heavy metal contamination levels using I_geo_ and potential ecological risk index (RI) values. 

Although spatial distribution and integrated heavy metal contamination have been extensively studied, current approaches for the assessment of soil heavy metal contamination in China are typically simplex ones. A simplex spatial distribution analysis and separate comprehensive index methods do not effectively and accurately reflect soil heavy metal contamination caused by mining and industrial activities and significantly affected by human factors [18,19]. We should also note that given human factors and the diffusibility of soil heavy metal contamination in mining and industrial gathering areas, conventional methods of soil partition and management based on soil function and land use are not enough to obtain accurate evaluation results on soil partitions and provide guidance for soil partition management in mining and industrial gathering areas.

A valid and applicable assessment method must be established to develop a clear understanding of soil contamination and to achieve effective partition assessment and soil management in typical industrial and mining areas. In the present study, an assessment method for soil heavy metal contamination caused by mining and industrial activities is developed based on the Nemerow index, I_geo_, and potential ecological RI. Moreover, a partition management method for mining and industrial gathering areas is developed through the cluster analysis method and spatial interpolation in the ARCGIS software environment. Soil heavy metal contamination is analyzed using an area in Tianjin, China, with a high number of mining activities as an example. Recommendations for soil partition management in the area are then proposed based on the monitoring of soil heavy metal contamination. The rest of the paper is organized as follows: Section 2 introduces the research method and the data sources for the study area. Section 3 presents the assessment results and discusses the effects of the main sources of pollution on soil quality. Section 4 discusses the conclusions.

## 2. Methodology

### 2.1. Study Area

The study area (118°07'–118°37' E, 38°33'–38°57' N) is located southeast of Tianjin, which is one of China’s largest industrial cities. Situated near Bohai Bay, the study area has a total land area of 1,113.83 km^2^. This area has a warm temperate semi-humid continental monsoon climate, with annual average temperature and precipitation levels of 14 °C and 600 mm, respectively. Given the rich reserves of oil and metal resources near the Bohai Bay, the study area has significant geographical and resource advantages. Since its establishment in 1979, the study area has been an important heavy industrial zone in Tianjin and even in the whole of China. The main industries developed in this area include petrochemical, metallurgy, and mining industries. According to the Tianjin New Town Overall Planning (2006–2020), this area is being developed as an important petrochemical industry base in northern China.

**Figure 1:**
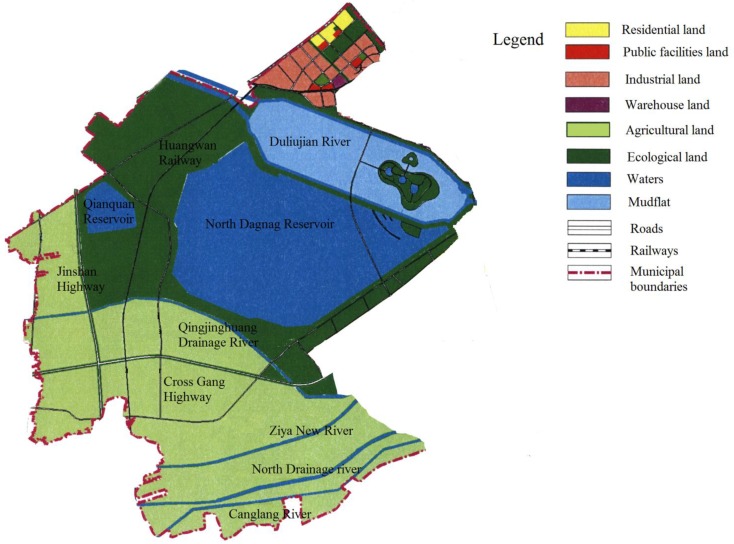
Landuse of study area.

The study area is a typical gathering and surrounding area for industrial and mining activities. As shown in Figure 1, industrial land, residential land and public facilities land in the northeast of the study area are mainly concentrated. To the south of the industrial areas, lie ecological land and water bodies that include the Duliujian River wetlands and North Dagang Reservoir. In the southwest of the study area, land is mainly used as agricultural land composed of villages. The heavy metal contamination in this area has an uneven distribution and is generally caused by human factors. Given its location in the river, sea, and land border zones, the ecological environment in this area is extremely sensitive and fragile. According to previous studies [20,21], heavy metal contamination occurs in various industrial and mining gathering areas and their vicinities, with rivers, farmlands, and coastal waters eventually suffering from different degrees of heavy metal contamination resulting from the discharge of pollutants into water bodies over long periods [22].

### 2.2. Sampling and Analysis

**Figure 2:**
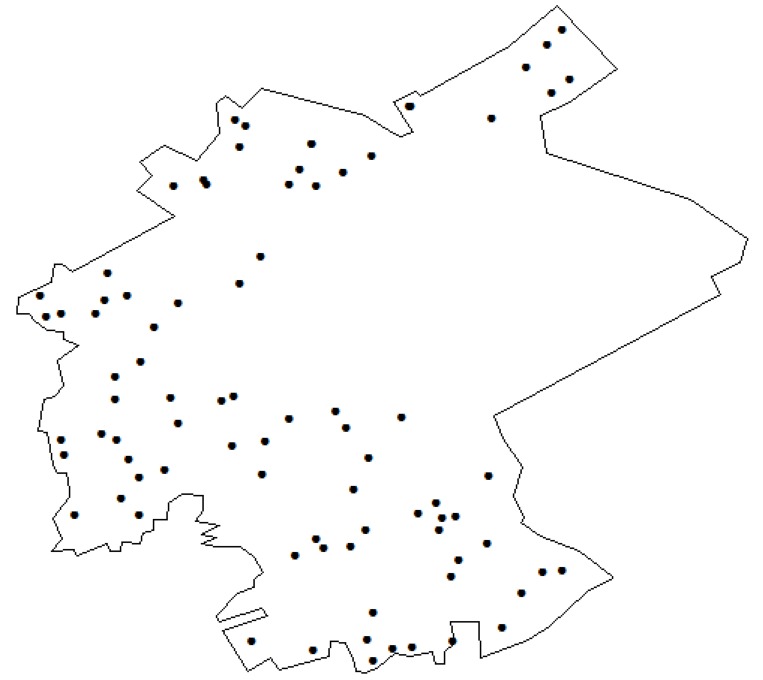
Soil sampling bitmaps.

Note: The black dots show the position of sampling points in the study area.

Soil samples were collected in the study area in 2005. As determined by systematic random grid sampling method, 85 census points were distributed according to soil type, topography characteristics, and distribution of contamination sources using a grid laying method (Figure 2). The large empty area in the sampling point map represents a water reservoir.

Eight common heavy metals, namely As, Cd, Cr, Cu, Ni, Pb, Zn, and Hg, were selected as monitoring targets. For pretreatment, the soil samples were air-dried at room temperature. Plant roots, organic residues, and visible intrusions in the samples were then removed. Finally, the samples were crushed, ground, and passed through a 0.85 mm sieve. Sample pH was measured in a deionized water suspension (1:2.5 v/v) after 1 h agitation using a Sartorius PB-10 instrument (Sartorius, Beijing, China ). Cd, Cu, Ni, Cr, Pb, and Zn concentrations were determined through inductively coupled plasma-atomic emission spectrometry. As and Hg concentrations were determined by atomic fluorescence spectrometry.

### 2.3. Assessment Method

#### 2.3.1. Cluster Analysis

Cluster analysis is a collective term covering a wide variety of techniques for delineating natural groups or clusters in data sets; it has been applied in many fields, including mathematics, statistics, and even metal industries to divide subjects into relatively homogenous clusters [23,24]. In the current study, cluster analysis of the eight kinds of heavy metals was performed to identify the relationship among the heavy metals and to group them according to their possible sources. Using the SPSS 19.0 software (IBM, Beijing, China ), this study selected relatively homogenous groups of variables through hierarchical cluster analysis. Before the start of the cluster analysis, heavy metal concentrations in each sampling point were standardized by means of Z-scores. The Euclidean distances for similarities were calculated by using the following formulae:

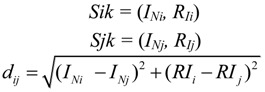
(1)

A dendrogram was constructed using the SPSS 19.0 software environment to assess the cohesion of readily seen clusters. The eight kinds of heavy metals were then divided into groups of different sources.

#### 2.3.2. Geo-Accumulation Index (I_geo_)

To make a comprehensive assessment of soil contamination, the Nemerrow index should be applied in this study. However, the traditional Nemerow index, which uses a single factor index method as the basis of the degree of contamination, couldn’t accurately reflect the heavy metal contamination with the impact of human behaviors. Therefore, in this paper, the geo-accumulation index, which could reduce the interference of human factors on assessment of soil contamination, is introduced to replace the traditional single factor index. I_geo_ was introduced by Müller [25] to assess metal pollution in sediments, and it has been applied in recent pollution studies to enable the qualitative assessment of soil heavy metal contamination [17,26]. I_geo_ is computed by Equation (2):

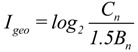
(2)
where C_n_ is the heavy metal concentration in the soil samples, and B_n_ is the geochemical background value in the average shale of the heavy metal element. To reduce the effect of parent rocks on heavy metal concentration and prominent heavy metal contamination in artificial soil, this study selected the geochemical background value as the average background value of elements in Chinese loess by referring to previous studies [19,27,28,29] and by considering the soil type of the study area (Table 1). The constant 1.5 compensates for the natural fluctuations of a given metal and for minor anthropogenic impacts. The seven classes of I_geo_ as proposed by Müller [25] are as follows: I_geo_ ≤ 0, uncontaminated (Class 0); 0 < I_geo_ ≤ 1, uncontaminated to moderately contaminated (Class 1); 1 < I_geo_ ≤ 2, moderately contaminated (Class 2); 2 < I_geo_ ≤ 3, moderately to heavily contaminated (Class 3); 3 < I_geo_ ≤ 4, heavily contaminated (Class 4); 4 < I_geo_ ≤ 5, heavily to extremely contaminated (Class 5); and I_geo_ > 5, extremely contaminated (Class 6).

**Table 1:** Geochemical background value (mg/kg).

Metals	As	Cd	Cr	Cu	Pb	Ni	Zn	Hg
Values	12.70	0.10	67.30	22.50	21.00	31.00	65.40	0.02

#### 2.3.3. Improved Nemerow Index (I_N_)

As I_geo_ could reduce the effects of parent rocks and prominent artificial effects on soil heavy metal contamination, it is suitable for the evaluation of soil heavy metal contamination in industrial and mining gathering areas. However, the evaluation of I_geo_ is only for a single heavy metal contaminant, thus this index cannot provide a comprehensive description of the contamination status of the study area. Accordingly, an evaluation based on the comprehensive index method is necessary. In this study, the traditional Nemerow index was improved by replacing the single factor index with I_geo_. The following Equation (3) was developed:

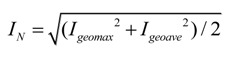
(3)
where I_N _is the comprehensive contamination index of a sample, I_geomax_ is the maximum I_geo_ value of such sample, and I_geoave _is the arithmetic mean value of I_geo_. To be consistent with I_geo_, the classification of I_N_ was adjusted based on the results proposed by Förstner [30]. This classification is as follows: 0 < I_N_ ≤ 0.5, uncontaminated (Class 0); 0.5 < I_N _≤ 1, uncontaminated to moderately contaminated (Class 1); 1 < I_N_ ≤ 2, moderately contaminated (Class 2); 2 < I_N_ ≤ 3, moderately to heavily contaminated (Class 3); 3 < I_N_ ≤ 4, heavily contaminated (Class 4); 4 < I_N_ ≤ 5, heavily to extremely contaminated (Class 5); and I_N_ > 5, extremely contaminated (Class 6).

#### 2.3.4. Potential Ecological Risk Index

As soil contaminated with heavy metals can enter the human body through various exposure approaches [31], highly toxic heavy metals in soil can cause serious ecological and health risks [32]. The excessive accumulation of heavy metals in agricultural soil can affect food quality and safety and further increase the morbidity of severe diseases, such as cancer, leukemia, and kidney or liver damage [33]. According to the Guidance on Working to Strengthen Food Safety from the Health and Family Planning Commission, Several Opinions about Deepen Rural Reform, and Accelerating the Modernization of Agriculture from the State Council of China, the monitoring of soil heavy metal contamination and the treatment of related soil pollution are important elements of food safety in China. In mining and industrial gathering areas, which have a high degree of heavy metal exposure, the assessment of potential ecological risks is necessary. To quantify the potential hazard from soil heavy metal contamination, the potential ecological RI was calculated in the present study as the sum of all the eight heavy metals. The potential ecological RI shows the sensitivity of the study area to soil heavy metal contamination and presents potential ecological risks.

Developed by Hakanson [34], the RI represents the toxicity of heavy metals and the response of the environment. It is calculated as:

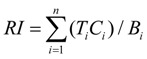
(4)
where RI is the monomial potential ecological risk factor, T_i_ is the toxicity factor of heavy metals based on the results of Hakanson [34] and Xu *et al.* [35], C_i_ is the practical concentration of metals in soil, and B_i_ is the background value for heavy metals. The toxic factors (T_i_) for the eight heavy metals are listed in Table 2. The adjusted valuation criteria for the potential ecological RI are as follows: RI ≤ 50, low risk; 50 < RI ≤ 100, moderate risk; 100 < RI ≤ 150, high risk; 150 < RI ≤ 200, very high risk; and RI > 200, extreme risk.

**Table 2:** Toxicity factors of heavy metals.

Metals	As	Cd	Cr	Cu	Pb	Ni	Zn	Hg
Values	10	30	2	5	5	5	1	40

#### 2.3.5. Spatial Analysis

Based on the assessment results for I_N_ and RI in the Spatial Analyst module of GIS 9.3 software environment (ESRI, Beijing, China), the spatial interpolation of the I_N_ and RI of 118 sampling points was performed by using the inverse distance weighted method. A spatial distribution map of I_N_ and RI was presented according to the interpolation results.

## 3. Results and Discussion

### 3.1. Overview of I_geo_

The main calculation results of I_geo_ in different soil samples are presented in Table 3. The average I_geo_ values of Hg, As, Ni, Cu, and Pb are less than 0. The average contamination level of these metals is categorized as Class 0, uncontaminated. The contamination level of Zn is categorized as Class 6, extremely contaminated. This result indicates that the soil in the study area has been mostly contaminated with Zn. Specific results and discussions on each heavy metal are provided in Section 3.3.

**Table 3:** Contamination index and level of different heavy metals.

Item	Hg	As	Ni	Cu	Zn	Pb	Cr	Cd
Maximum	1.782	−0.335	−0.290	0.173	6.079	−0.325	0.652	1.860
Minimum	−4.967	−1.792	−1.662	−1.402	4.336	−1.635	−0.643	−0.862
Average	−0.450	−0.901	−0.653	−0.467	5.349	−0.761	0.234	0.060
Average contamination level	0	0	0	0	6	0	1	1

### 3.2. Cluster Analysis

**Figure 3:**
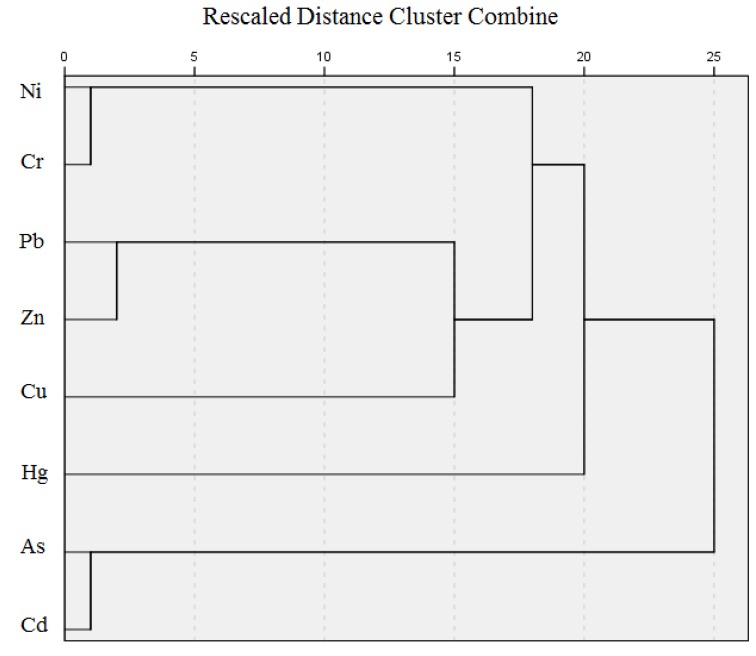
Dendrograms produced by hierarchical clustering.

Cluster analysis was performed to further divide the clusters of heavy metal contaminants and to analyze possible sources. The results are illustrated in a dendrogram in Figure 3. The distance cluster represents the degree of association among heavy metals; a low distance cluster value indicates a significant relationship [36]. As shown in Table 3, the first group includes Ni and Cr, the contamination levels of which are relatively low. According to previous studies [17,37], this group of heavy metals can be associated with atmospheric deposition because Cr enters soils mainly through deposition and Ni is a heavy metal that draws attention to anthropogenic sources, such as mechanical wear, welding, processing, and oil combustion. As the contamination levels of these heavy metals are low, they are unlikely to be directly emitted by relative industries. The second group includes Pb, Zn, and Cu, which are commonly associated metals and are generally detected in industrial wastewaters. Given the high contamination level of Zn (Table 3), emissions as well as industrial wastewater and waste gases are the main contamination sources of this group. The third group includes Cd and As, which are mainly from the mining and smelting of non-ferrous metals; coal and oil combustion are also important sources of these heavy metals [38]. The last group includes only Hg, which mainly comes from chlor-alkali production, plastics, batteries, and electronics.

### 3.3. Analysis of Heavy Metals

#### 3.3.1. Cadmium

**Figure 4:**
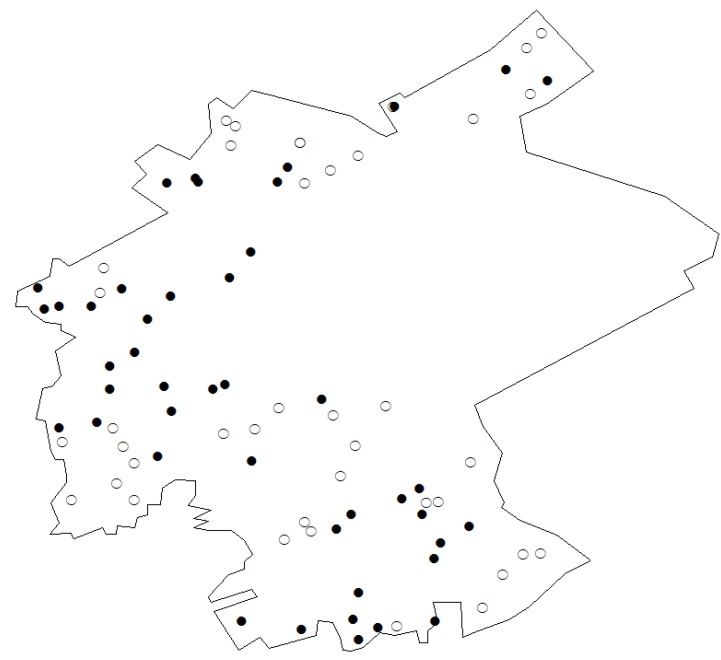
Distribution of Cd points.

Note: The black and white dots show the sampling points with high I_geo_ (I_geo_ > 0) and the remaining, respectively.

Cd exhibits highly adverse effects on human health and ecological safety. In the study area, the average I_geo_ value of Cd is 0.060, which indicates that the overall level of Cd contamination in the study area can be categorized as uncontaminated to moderately contaminated. The highest I_geo_ value of Cd is 1.860. In addition, 44 sample points have I_geo_ values exceeding 0 and account for 51.76% of all 85 sample points. As shown in Figure 4, points with high I_geo_ (I_geo_ > 0) have a scattered distribution and do not show any central tendencies. 

The separate distribution and diffusibility of Cd contamination can be determined from the distribution results. As a catalyst and an intermediate product, Cd is widely used in electroplating, chemical, electronics, non-ferrous metals, and nuclear industries. According to the Census of Pollution Sources (2005) [39], five electroplating enterprises and six non-ferrous metal smelting enterprises operate in the study area. These industries are main sources of Cd contamination. The mobility of Cd shows that vehicles and coal and oil combustion are also sources of Cd contamination in the study area.

#### 3.3.2. Chromium

Cr has been widely recognized as a heavy metal that causes serious harm to human health and is known to have carcinogenic and teratogenic effects. In the study area, the average I_geo_ value of Cr is 0.234, which indicates that the overall level of Cr contamination in the study area ranges from uncontaminated to moderately contaminated. The highest I_geo_ value of Cr is 0.652. Although the overall contamination level in the study area is relatively low, the I_geo_ value of Cr for up to 75 sample points exceeds 0. These sample points account for 88.24% of the total. As an important material in metal processing, electroplating, and leather industries, Cr has been widely used in the study area, which is a typical industrial and mining gathering area. As a result, Cr contamination was observed here. According to the Census of Pollution Sources (2005) [39], 42 metal processing enterprises and six electroplating enterprises operate in the study area. These industries are the main sources of Cr contamination. As indicated by the wide distribution of this heavy metal, the diffusibility of Cr contamination, which spreads through the industrial wastewater discharged into the ground, should be noted. 

#### 3.3.3. Zinc and Lead

Zn has been extensively documented as one of the most readily mobile and concerning elements, particularly because of its toxic and carcinogenic effects [17]. The results of the present work show certain differences between Zn and Pb. The average I_geo_ value of Pb is only −0.761, which indicates low contamination level. By contrast, the highest I_geo_ value of Zn is 6.079, and its average I_geo_ value is 5.349; its I_geo_ values for almost all sample points exceed 5 (except the lowest 5 points). These values indicate the high contamination intensity and strong diffusivity of Zn. 

As shown in the cluster analysis results, Pb and Zn belong to the same group and come from the same industrial emission sources. However, the scope of use of these two metals differs. In addition to mining and smelting, industries such as paper, machinery, metal manufacturing, and zincification are sources of Zn contamination. Pb contamination is mainly caused by the use of Pb products and non-ferrous metallurgy. According to the Census of Pollution Sources (2005) [39], four paper-making enterprises, two galvanized metal and Zn smelting enterprises, three machinery manufacturing enterprises, and 42 metal and metal product manufacturing enterprises operate in the study area. A large number of these industries are main sources of Zn contamination. Meanwhile, only a small number of industries use Pb as a raw material. The main sources of Pb contamination are six non-ferrous metal enterprises. Vehicles are also sources of Zn and Pb contamination because the production and use of car tires cause Zn contamination and oil combustion emits waste gases with Pb.

#### 3.3.4. Mercury

**Figure 5:**
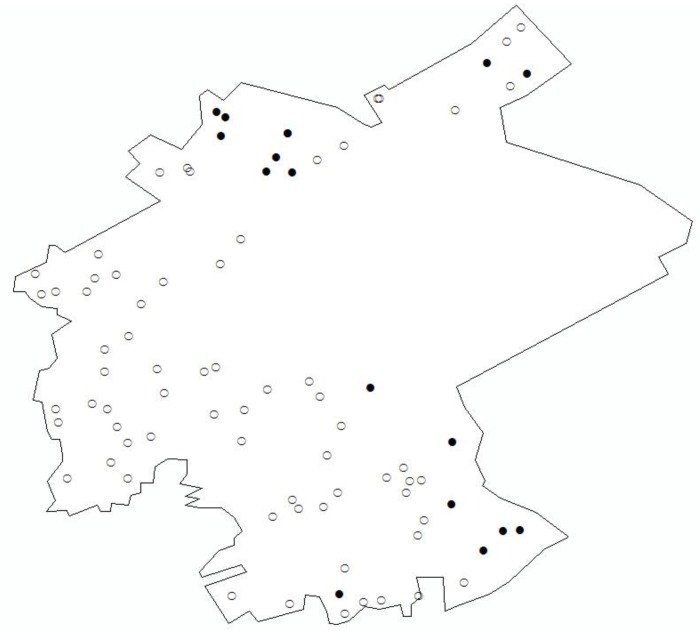
Distribution of Hg points.

Note: the black and white dots show the sampling points with high I_geo_ (I_geo_ >0)and the remaining, respectively.

Hg from chlor-alkali production waste, plastics, batteries, electronics, and waste medical devices can cause harm to the ecosystem and the human body in the form of various compounds. In the study area, the average I_geo_ value of Hg is −0.450, which indicates that the overall level of Hg contamination in the study area is classified as uncontaminated. However, the highest I_geo_ value of Hg is 1.782 for 19 sampling points whose I_geo_ is higher than 0. As shown in Figure 5, Hg shows relatively low contamination levels compared with Cr, Cd, and Zn. Nevertheless, the hazards posed by such contamination should not be underestimated, especially in the areas with a high concentration of sampling points with high I_geo_ values. According to the Census of Pollution Sources (2005) [39], two plastic enterprises and one electronics enterprise are the main sources of Hg contamination in the study area. 

#### 3.3.5. Arsenic, Copper, and Nickel

The average I_geo_ values of As, Cu, and Ni are ‒0.901, ‒0.467 and ‒0.653, respectively, which are all less than 0. For As, Cu, and Ni, percentages of I_geo_ that are higher than 0 are very low, which are 0, 1.10% and 3.30%, respectively. The distribution of the sampling points with relatively high I_geo_ values is scattered. These sampling points are not concentrated in any area. According to the Census of Pollution Sources (2005) [39], the study area does not have mining and smelting enterprises, which are main sources of As, Cu, and Ni contamination. In addition, the study area does not have alloy steel enterprises, which use Ni as an important raw material. As mining and smelting, which use chemical fertilizers containing as, are also not set up in the study area. The cluster analysis results show that atmospheric depositions from mechanical wear, welding, and the processing and combustion of oil are the main sources of Ni contamination. Meanwhile, emissions from industrial wastewater containing a small amount of Cu are the main resources of Cu contamination. Results show the contamination is mainly caused by oil and coal combustion.

### 3.4. Improved Nemerow Index

**Figure 6:**
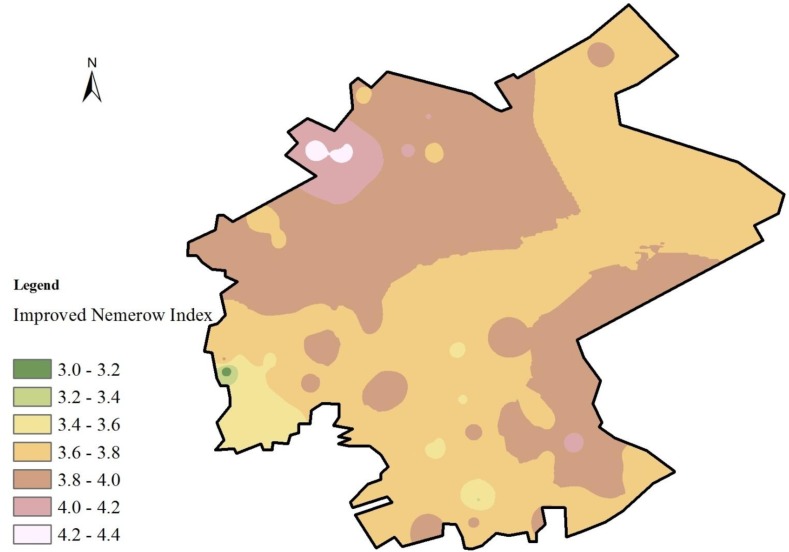
Spatial distribution map of I_N_.

To quantify the soil heavy metal contamination in the study area, the improved Nemerow index (I_N_), which describes the integrated contamination levels, was calculated as the sum of the eight heavy metals (Figure 6). The I_N_ results at all sampling points indicate the following. (1) In general, the I_N_ value of the study area is between 3 and 5, given the maximum and minimum values of 4.311 and 3.079. This finding reveals that the overall level of heavy metal contamination in the study area is between heavily and extremely contaminated, which indicates serious heavy metal contamination. (2) The I_N_ values of only seven sampling points exceed 4, which indicates that the contamination level is approaching extremely contaminated. The distribution of the sampling points with high I_N_ values is similar to that of the sampling points of Hg with high I_geo_ values. Given the high toxicity of Hg, the distribution of Hg contamination levels has a significant effect on the integrated contamination level. (3) The uneven distribution of the contamination degree is another important feature of heavy metal contamination in the study area. As shown in Figure 6, the most polluted areas (I_N_ > 3.8) are not distributed in any concentrated area.

Soil heavy metal contamination in mining and industrial gathering areas can be caused by human behavior. Atmospheric deposition and groundwater contamination, which result from prevailing wind direction changes, fossil fuel combustion, and irregular discharge of industrial sewage in the study area, are also important causes of soil heavy metal contamination. The reasons for the irregular distribution of soil heavy metal contamination in the study area, as well as in other industrial and mining gathering areas, are intricate. The traditional method for soil partition, which is based on soil function and land use [40,41], can hardly adapt to the needs of industry and mining gathering areas. 

### 3.5. Potential Ecological Risk Analysis

**Figure 7:**
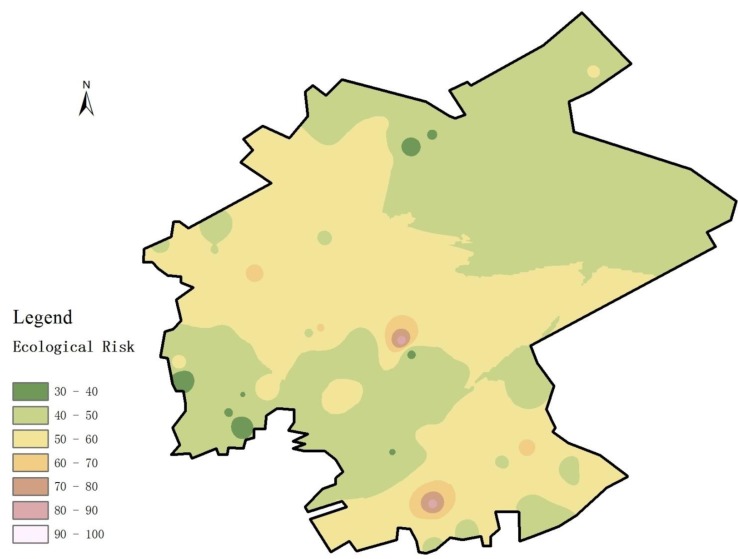
Spatial distribution map of RI.

As a complement to the study on integrated contamination levels, the potential ecological risk of soil heavy metal contamination in the study area was quantified by calculating the potential ecological RI, which describes the ecological risk level in the area (Figure 7). The RI results for all sampling points reveal the following. (1) The RI of the study area for all sampling points is from 30 to 100, which shows an overall ecological risk level ranging from low to moderate. A total of 44 sampling points have RI values lower than 50, which indicates that the ecological risk level in a large portion of the study area is low. (2) The maximum RI reaches 84.567, with only two sampling points exceeding 80. Figure 7 shows that moderate ecological risk (RI > 50) can be observed mainly in the central and southern regions. The figure also indicates a certain degree of uneven distribution, especially in the southwestern regions where the levels of ecological risk follow a criss-cross pattern. (3) RI distribution does not show the same trends as the I_N_ results. The relationship between contamination risk and contamination levels should thus be further studied.

## 4. Conclusions

Using the Nemerow and geo-accumulation indexes as basis, this study introduces an improved Nemerow index method for the assessment of soil heavy metal contamination in industrial and mining gathering areas. The analysis of a typical industrial and mining gathering area in Tianjin (China), provides important information about the distribution of As, Cr, Cd, Cu, Ni, Pb, Zn, and Hg in the area. Cluster analysis shows that Ni and Cr have a close relationship, and their presence may be mainly related to vehicle emissions and atmospheric deposition. Zn poses an extremely serious pollution threat because it shows the highest contamination level among the eight heavy metals in the study. The contamination levels of the other seven heavy metals are relatively low. The results of the improved Nemerow index and the spatial interpolation indicate that the overall level of heavy metal contamination in the study area ranges from heavily to extremely contaminated and that the integrated contamination level shows an uneven distribution. Potential ecological risk analysis also shows an uneven distribution of ecological risk in the study area. The overall ecological risk level ranges from low to moderate.

Understanding the degree, scale, and sources of heavy metal contamination is essential for environmental management. It is also important in reducing risks to human health, ensuring food safety, and managing contaminated soil. This study presents important information about soil heavy metal contamination in industrial and mining gathering areas and provides systematic methods for the assessment of heavy metal contamination and the identification of contamination sources in these areas. In future studies, the partition of study areas based on contamination levels should be considered as a research focus. Spatial analysis methods such as spatial overlay must be applied extensively, and human health risk should be seriously considered in partition studies.
